# Curve matching to predict growth in patients receiving growth hormone therapy: An interpretable & explainable method

**DOI:** 10.3389/fendo.2022.999077

**Published:** 2022-10-05

**Authors:** Paula van Dommelen, Lilian Arnaud, Ekaterina Koledova

**Affiliations:** ^1^ Department of Child Health, The Netherlands Organization for Applied Scientific Research (TNO), Leiden, Netherlands; ^2^ Global Digital Health, Ares Trading S.A. (an affiliate of Merck KGaA), Eysins, Switzerland; ^3^ Global Medical Affairs Cardiometabolic & Endocrinology, Merck Healthcare KGaA, Darmstadt, Germany

**Keywords:** curve matching, growth disorders, growth hormone, prediction model, paediatric, growth hormone deficiency, small for gestational age

## Abstract

Curve matching may be used to predict growth outcomes using data of patients whose growth curves resemble those of a new patient with growth hormone deficiency (GHD) and those born small for gestational age (SGA). We aimed to investigate the validity of curve matching to predict growth in patients with GHD and those born SGA receiving recombinant human growth hormone (r-hGH). Height data collected between 0–48 months of treatment were extracted from the easypod™ connect ecosystem and the easypod™ connect observational study. Selected patients with height standard deviation scores (HSDS) [-4, <-1] and age [3, <16y] at start were included. The ‘Matching Database’ consisted of patients’ monthly HSDS obtained by the broken stick method and imputation. Standard deviation (SD) was obtained from the observed minus the predicted HSDS (error) based on matched patients within the ‘Matching Database’. Data were available for 3,213 patients in the ‘Matching Database’, and 2,472 patients with 16,624 HSDS measurements in the observed database. When ≥2 HSDS measurements were available, the error SD for a one-year prediction was approximately 0.2, which corresponds to 1.1 cm, 1.3 cm, and 1.5 cm at 7, 11, and 15 years of age, respectively. Indication and age at treatment start (<11 vs ≥11 years) had a small impact on the error SD, with patients born SGA and patients aged <11 years at treatment start generally having slightly lower values. We conclude that curve matching is a simple and valid technique for predicting growth in patients with GHD and those born SGA.

## Introduction

The use of recombinant human growth hormone (r-hGH) therapy to treat growth disorders in children is well established (1). However, patients often fail to reach their full height potential, with suboptimal adherence being one contributory factor (2). Furthermore, there is high individual variability in the growth response to r-hGH treatment, which can also be caused by the underlying growth disorder (3). Distinguishing between the relative contributions of the aforementioned factors is important information for clinical decision making.

The availability of clinical data from large observational studies of r-hGH in pediatric patients with growth disorders (4, 5) has provided the opportunity to analyze the factors that determine responsiveness to r-hGH treatment. This has led to the development of prediction models that demonstrate potential in predicting future growth in patients receiving r-hGH for growth disorders. This not only supports healthcare professionals (HCPs) to identify poor responders and to individualize treatment to optimize growth outcomes, but also allows patients with growth disorders the opportunity to see the expected effect of their r-hGH treatment. Flexible prediction models based on clinical practice and real-world data could provide relevant goals and expectations setting for the families towards achievable outcomes.

The most frequently used method of deriving growth prediction models has been multiple linear regression (6–12); however, the non-linear technique of empirical curve fitting (13, 14), and the machine learning technique of Artificial Neural Networks have also been applied (15–17). While these models can accurately predict growth, there are several challenges in integrating them into clinical practice to guide HCPs in their decision-making. Some of these challenges are that considerable information from patients is needed, such as age at puberty onset, birth weight, and previous year height velocity (this can be determined less well with irregular observation times). In addition, the prediction models are only applicable at fixed times (at treatment start and annually) while, in reality, patients may not attend periodic visits or the time at which they attend the visit can differ between patients for one or more months. A ‘one-size-fits-all’ approach (group-level models) thus does not work for all patients or healthcare systems. Furthermore, prediction models are known to underestimate relatively low predictions and overestimate relatively high predictions (18, 19); the accuracy of a high prediction of height velocity is less than the accuracy of a low prediction (19), and this information is generally not presented to HCPs, once again hindering the implementation of prediction models in clinical practice. Finally, in order to be adopted by HCPs, the applied methods should be interpretable (20) and explainable in order for HCPs to understand, trust, and use the results. Therefore, there is a need for simple prediction methods that are (1) easy to understand (2), in line with HCPs’ current clinical workflows, and (3) do not increase workload or require additional time that, in reality, is not available in routine clinical practice.

A method that may meet these requirements is curve matching (21). To apply this method a longitudinal database is needed with a large amount of children’s longitudinal growth data that forms the basis of developing growth curves. This database can be used to identify growth curves (e.g. height) of children that are similar to the growth curve of a new child up until their current visit. The growth curves of these ‘matched’ children can then be added to the growth chart of the new child to visualize how this child is expected to grow in the future. For patients receiving r-hGH for GHD and SGA, we have applied this concept using height standard deviation (HSDS) during treatment from real-world patients as a curve matching technique to visualize future catch-up growth. In addition, we have also developed a method to predict the growth curve based on these matched patients.

In this respect, the objective of our study was to investigate the validity of curve matching to predict growth in patients with growth hormone deficiency (GHD) and those born small for gestational age (SGA) receiving r-hGH. We hypothesized that the curve matching technique may be a new and accurate technique for predicting growth in patients with GHD and those born SGA.

## Methods

### Original data

An observational study was performed where we included data from patients aged <19 years from two sources that partly overlap: the easypod™ connect ecosystem (extraction date: 25 February 2022) and the easypod™ connect observational study (ECOS) (4). The ECOS was an open-label, observational, longitudinal study conducted in 24 countries between 2010 and 2016, enrolling children receiving treatment with r-hGH. Eligible patients attended one baseline visit followed by 1–4 visits per year, according to local routine clinical practice. Planned duration of follow-up was at least every 6 months for up to 5 years (4). Within the easypod™ connect ecosystem, 4,070 patients with 20,535 height measurements were available. An additional 5,628 height measurements were available from the ECOS for 607 patients from the easypod™ connect ecosystem. Also, 1,754 patients with 13,889 height measurements from the ECOS, who were not part of the easypod™ connect ecosystem, were included. We then selected measurements between 0–54 months of treatment. In total, data for 5,792 patients with 33,760 height measurements were available. Furthermore, we selected patients with ≥2 height measurements during 0–54 months of treatment, which resulted in data for 4,901 patients with 32,869 height measurements.

### Cleaning

World Health Organization (WHO) growth references were used to calculate HSDS (22, 23). We selected measurements with a HSDS between [>-7, <2 SD] of all available measurements during treatment: 4,898 patients with 32,789 height measurements were identified. Height measurements which decreased by ≥2 cm (taking measurement error of approximately 0.3 cm*3 SD=0.9 cm on either side of each height measurement into account as a first cleaning step) were excluded (67 height measurements were removed). We then applied the broken stick method (24), with Kasim-Raudenbush sampler with a linear mixed model using a second-order linear B-spline. The broken stick method approximates each patient’s HSDS trajectory by a series of connected straight lines between breakpoints. The breakpoints divide the time on treatment axis into consecutive intervals common to all patients. The broken stick method is a special case of the linear mixed model with subject as the grouping factor. The main assumptions are: Subjects are exchangeable, trajectories between two breakpoints are all straight, random effects follow a multivariate normal distribution, and unobserved data are missing at random. Although the number and timing of measurements differed by patient, these assumptions allowed us to transform the irregular observation times into estimates of repeated measures of three-monthly HSDS between 0–48 months (these were the chosen breakpoints) of treatment for all patients. Excluding patients with fewer measurements would introduce biased estimates. Outliers were defined as residuals of HSDS ≤-0.5 and ≥0.5 and were excluded from analysis (71 height measurements were removed). In total, 4,896 patients (2,642 GHD, 697 SGA, 235 Turner syndrome [TS], 138 other, 1,184 unknown) with 32,651 height measurements were available for analysis.

### Methodology

We applied multiple imputation by chained equations (25) to impute missing values of indication (24% missing). Within the imputation model, we included several other clinical parameters and background characteristics: three-monthly HSDS between 0–48 months of treatment obtained from the broken stick model, region (America, Asia-Pacific, Europe) (0% missing), age at treatment start (0% missing), sex (0% missing), parental height (46% missing), birth weight (47% missing), birth length (59% missing), gestational age (52% missing), and three-monthly dose (mg/kg/week) (10–76% missing). In total, five imputed datasets were obtained. We then applied linear interpolation to obtain monthly HSDS between 0–48 months of treatment.

### Selection criteria

We selected the indications GHD and SGA (because of their large sample sizes) based on observed and imputed data with an agreement of indication in at least four out of five imputed datasets. In total, 4,345 patients (3,449 GHD, 896 SGA) were available. We then selected: 1) HSDS at treatment start between [-4, <-1 SD] (2,886 GHD, 799 SGA); 2) growth curves with a yearly increase (HSDS>0) between 0–48 months (2,590 GHD, 736 SGA); and 3) age at treatment start between [3, <16 years] (2,487 GHD, 726 SGA). This ‘Matching Database’ included GHD and SGA patients (n=3,213) with monthly HSDS between 0–48 months on treatment. We did not differentiate between patients with GHD and those born SGA, because their growth trajectories were comparable; mean (SD) HSDS were -2.3 (0.7) at 0 months and -1.0 (0.8) at 45–48 months in patients with GHD, and -2.5 (0.6) at 0 months and -1.1 (0.8) at 45–48 months in patients born SGA.

### Weighing score

A weighing score was calculated for each patient within the ‘Matching Database’ based on the extent to which the same growth curves occurred in the database. This weighing score was defined as the number of good (defined as a residual SD <0.03) matches with the other patients in the database + 1 (the patient itself) divided by the total number of patients.

### Matching technique

**Figure 1:**
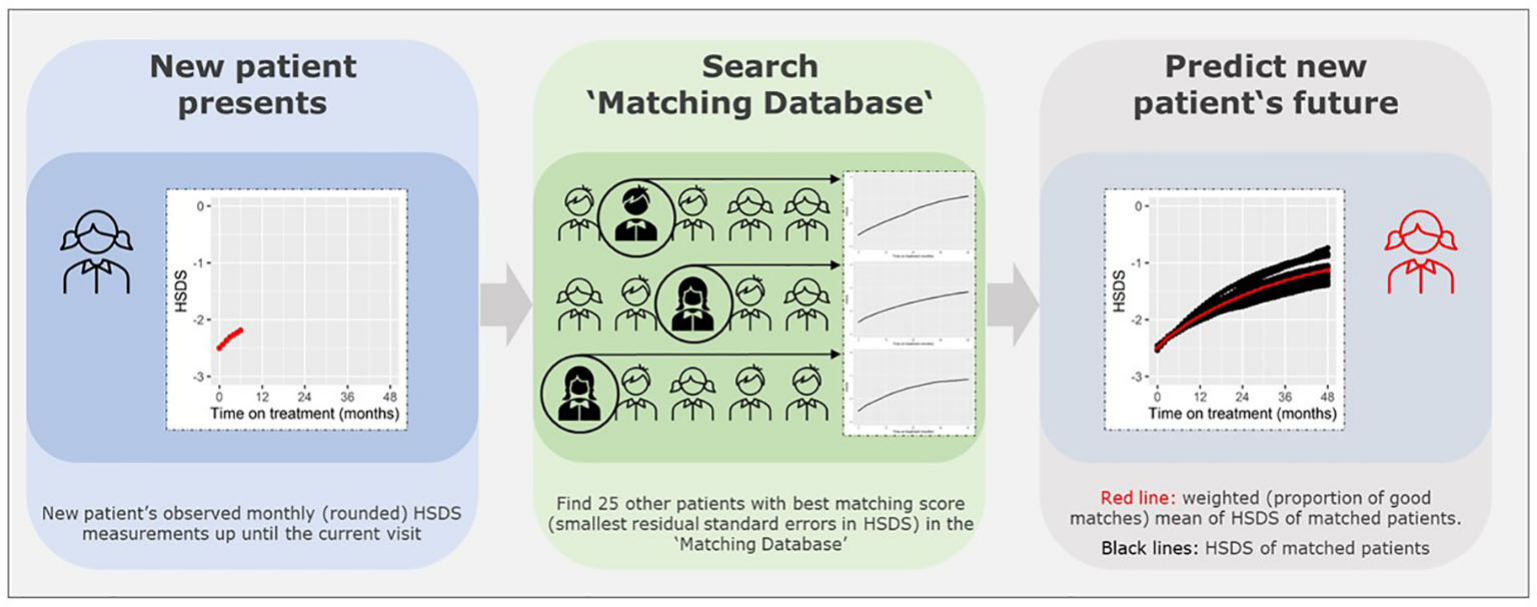
Technique to find matches based on similar growth curves.

**Figure 1** presents a visual presentation of the curve matching technique. To match a patient and predict growth of HSDS after a visit, we need to investigate the difference between the patient’s observed monthly (rounded) HSDS measurements up until a visit, and the monthly HSDS measurements up until that visit for each individual patient in the ‘Matching Database’ by calculating the residual standard errors in HSDS (note: divided by n if number of measurements <3). We then select 25 patients with the smallest residual standard errors in HSDS.

**Figure 2:**
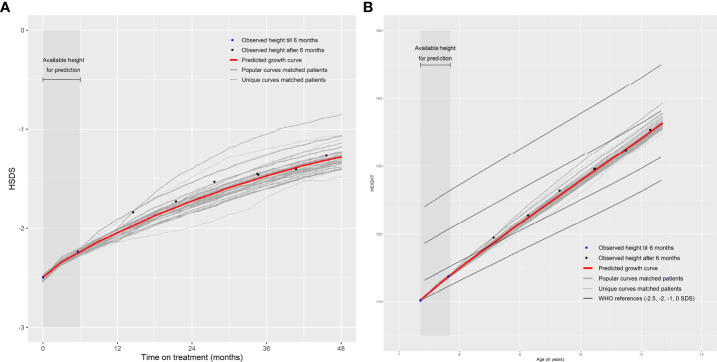
Good prediction of future growth comparing **(A)** observed HSDS with predicted HSDS and **(B)** observed height with predicted height, based on 25 matched patients.

**Figure 3:**
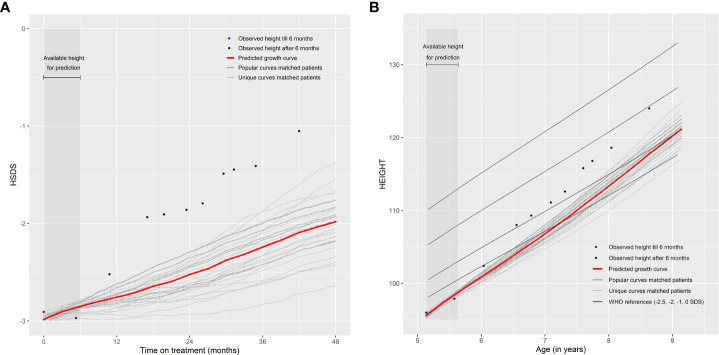
Poor prediction of future growth comparing **(A)** observed HSDS with predicted HSDS and **(B)** observed height with predicted height, based on 25 matched patients.

Note that the patients themselves are removed from the ‘Matching Database’ before matching, otherwise they will be matched with themselves. The weighted (by the weighing score) mean of monthly HSDS after that visit of the 25 matched patients can then be calculated. The individual growth curves of these 25 matched patients and the weighted mean curves can be visualized in a growth chart. A total of 25 patients were chosen because fewer patients would cause less stable weighted means and more patients would require a sufficient number of strong matches and complicate the visualization of the growth curves. One can highlight the curves (large line width) that are more popular (high weighing score and, therefore, such a growth curve is more common in the ‘Matching Database’) and make unique curves (low weighing score and, therefore, such a growth curve is unique in the ‘Matching Database’) less visible (lower line width). The weighted mean curve can be displayed in a different color (for example, red). One can also see the accuracy of the prediction; if these 25 matched growth curves are close to each other then it is very likely that the patient will follow this growth curve in the future; conversely, if the growth curves are far apart, the patient’s growth curve is difficult to predict. For example, **Figures 2A, B** provide a visual representation of a new patient where the predicted growth curve provides a good estimation of future growth, while **Figures 3A, B** present another patient for whom the predicted growth curve provides a poor estimation. **Figures 2B, 3B** are calculated based on HSDS converted to height using the age and sex of the new patient. Within the figures, HSDS up until 6 months of treatment were used to match patients. When we plot the observed future HSDS or height measurements (which are unknown at that moment in time), we find that the predicted growth curve is close to the observed HSDS in **Figure 2**. Within **Figure 3**, the HSDS up until 6 months of treatment of the patient is almost stable, which results in matches of patients with a relatively low catch-up growth. After 6 months of treatment the patient unexpectedly, and unlike other patients, shows a strong catch-up growth and, therefore, the predicted growth curve is much lower than the observed HSDS because this new information was not taken into account. However, when the patient has a visit at around 12 months after treatment start, curve matching can once again be applied using all HSDS up until 12 months, and patients who have a stronger catch-up growth will be matched with this patient. This in turn results in a predicted curve that is closer to the observed HSDS in the future. Importantly, such curve matching can be applied at each visit, taking all information available at that point of time into account.

### Validation

For the validation of the curve matching technique, we used the original cleaned data and applied the same selection criteria as mentioned in the paragraph ‘Selection criteria’. In total, 2,472 patients with observed (no broken stick) height measurements and known (no imputation) disorders (1,897 GHD, 575 SGA) were available.

The growth curve of each patient can be matched with other patients’ growth curves. However, to validate the curve matching technique, we investigated five groups with height measurements within certain time intervals in order to compare the actual observed measurements after the prediction minus the expected growth curve based on earlier measurements.

The selected five groups comprised:

1. Selection of patients with HSDS at treatment start known and ≥1 after start for validation (n=1,356 patients [1,062 GHD, 294 SGA]) to predict growth when only HSDS is known at start;

2. Selection of patients with known HSDS at start and ≥1 measurement at 1–3 months and ≥1 after 3 months for validation (n=689 patients [547 GHD, 142 SGA]) to predict growth when HSDS is known up until 1–3 months of treatment;

3. Selection of patients with known HSDS at start and ≥1 measurement at 4–6 months and ≥1 after 6 months for validation (n=843 patients [681 GHD, 162 SGA]) to predict growth when HSDS is known up until 4–6 months of treatment;

4. Selection of patients with known HSDS at start and ≥1 measurement at 9–12 months and ≥1 after 12 months for validation (n=853 patients [686 GHD, 167 SGA]) to predict growth when HSDS is known up until 9–12 months of treatment;

5. Selection of patients with known HSDS at start and ≥1 measurement at 7–12 months and at 13–24 months, and ≥1 after 24 months for validation (n=630 patients [502 GHD, 128 SGA]) to predict growth when HSDS is known up until 13–24 months of treatment.

Within each group, we developed boxplots by months on treatment with the distance between the observed and the predicted HSDS (error) based on the weighted mean curves of the 25 matched patients as the prediction method. Within the boxplot, a box is presented from the first to the third quartile. A vertical line goes through the box at the median. The whiskers go from each quartile to the minimum and maximum. Also, we calculated the SD of the error by months on treatment. Categories by months of treatment were defined as 4–9, 10–14, 15–20, 21–27, 28–32, 33–39, and 40–48 months. R Version 4.0 with MICE, AGD, and broken stick packages were used to analyze the data.

## Results

**Table 1** shows the clinical parameters and background characteristics of the observed data and the ‘Matching Database’. In total, 2,472 patients with 16,624 measurements within the observed data and 3,213 patients with monthly measurements within the ‘Matching Database’ were available. Within the observed data the following measurements were available: 1,377 measurements at treatment start; 5,467 between 1–11 months; 4,789 between 12–23 months; 3,134 between 24–35 months; and 1,857 between 36–48 months. The number (and timing) of measurements per patient varied from 2–31, with an average number of measurements of 7. The majority of patients were boys (64%) with the GHD indication (77%), who were, on average, nearly 10 years of age when they started treatment, and a mean height at treatment start of -2.4 SD. Specifically, the mean (SD) age at treatment start was 9.9 years (3.2 years) in GHD patients and 8.6 years (3.1 years) in patients born SGA.

**Table 1:** Clinical parameters and background characteristics of the observed data (n = 2,472) and the ‘Matching Database’ (n = 3,213).

Parameter	Observed data		Matching Database	
	Mean (SD)	N (%)	Mean (SD)	N (%)
Sex				
Girls		895 (36)		1,186 (37)
Boys		1,577 (64)		2,027 (63)
Indication				
GHD		1,897 (77)		2,487 (77)
SGA		575 (23)		726 (23)
Region				
America		715 (29)		845 (26)
Asia-Pacific		490 (20)		636 (20)
Europe		1,267 (51)		1,732 (54)
Birth length, cm	48 (4.0)		48 (4.1)	
Birth weight, g	2,799 (695)		2,825 (678)	
Gestational age, weeks	38 (3.1)		38 (2.8)	
Height father, cm	172 (7.1)		172 (7.2)	
Height mother, cm	159 (6.7)		159 (6.7)	
Age at treatment start, years	9.6 (3.2)		9.8 (3.2)	
HSDS at treatment start	-2.4 (0.7)		-2.3 (0.7)	

**Figure 4:**
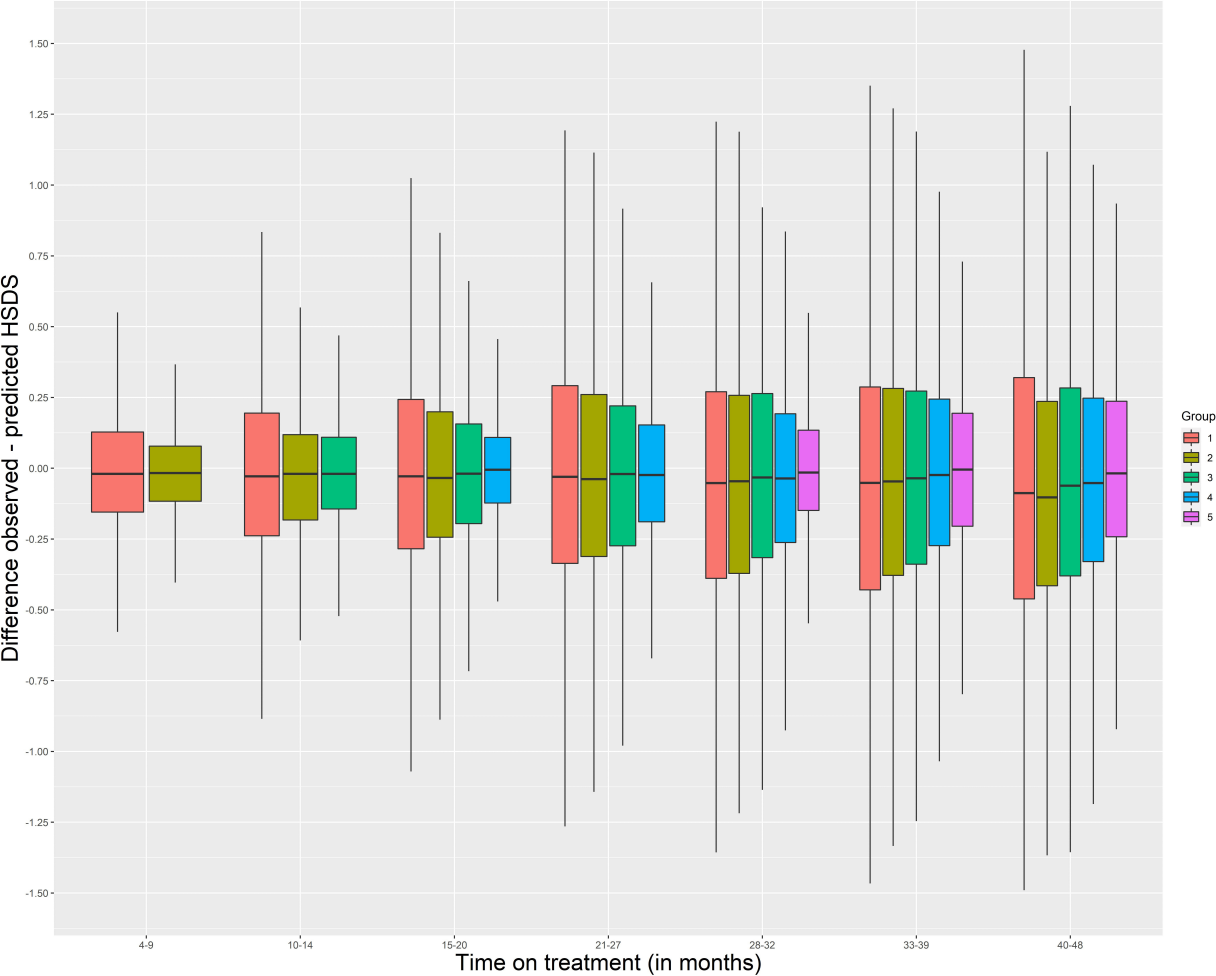
Boxplots by months on treatment using the distance between the patients’ observed minus the predicted HSDS based on the weighted mean curves of 25 matched patients Group 1: HSDS at start known (n = 1,356) Group 2: HSDS at start known and ≥1 measurement between 1–3 months (n = 689) Group 3: HSDS at start known and ≥1 measurement between 4–6 months (n = 843) Group 4: HSDS at start known and ≥1 measurement between 9–12 months (n = 853) Group 5: HSDS at start known and ≥1 measurement between 7–12 months and between 13–24 months (n = 630) HSDS, height standard deviation score.

**Figure 4** shows boxplots by months on treatment using the distance between the patients’ observed minus the predicted HSDS based on the weighted mean curves of 25 matched patients. This figure shows that an additional measurement in the first three months of treatment on top of a measurement at treatment start improved the accuracy of the growth prediction (Group 2 vs Group 1). The same holds for an additional measurement between 4–6 months (Group 3), 9–12 months (Group 4), and between 7–12 months and 13–24 months (Group 5).

The error SDs were: 0.3 in Group 1 and 0.2 in Group 2 at 12 months (range 10–14) of treatment; 0.4 in Group 2, 0.3 in Group 3, and 0.2 in Group 4 and 5 at 24 months (range 21–27); 0.4 in Group 4 and 0.3 in Group 5 at 36 months (range 33–39); and 0.3 at 48 months (range 40–48). Indication and age at treatment start (<11 vs ≥11 years) only had a small impact on the error SD, with patients born SGA and patients aged <11 years at treatment start generally having slightly lower values. However, the error mean was approximately 0.2 SD higher after 24 months of treatment in patients who were ≥11 years at treatment start, showing that the method expected, on average, a lower catch-up growth. Matching with patients who have a similar indication (GHD or SGA) showed that the error SDs were almost similar or, for several groups, slightly higher compared to matching with all patients.

## Discussion

### Curve matching to predict future growth

Large datasets of growth data are invaluable to predict growth outcomes in children with GHD and SGA receiving treatment with r-hGH. Curve matching can support HCPs, patients, and caregivers in this regard by providing a simple and understandable method that takes currently available information from large data sets into account and is comparatively easy to implement in clinical practice. In the presented study, the availability of big real-world height data enabled us to investigate the validity of curve matching to visualize and predict future growth in patients with GHD and those born SGA who were prescribed r-hGH. We matched new patients with other patients included in the data who displayed similar growth curves and investigated the growth of these matched patients throughout treatment. This information was then used to predict future growth for the new patient. Importantly, predictions can be recalibrated at each visit by considering all available information (from the patient and in the database) at that specific point in time. Therefore, at each visit the predicted HSDS based on the information from the previous visit minus the observed HSDS can be helpful to visualize the current status, with the new prediction showing the potential long-term effects of growth hormone therapy. This may provide meaningful information to patients and their caregivers to improve adherence. If adherence, and therefore growth, is improved, then recalibration becomes important and this needs to happen if the growth trajectory changes. Moreover, if adherence is deemed acceptable, then other factors need to be considered as to why growth is impaired or slower than expected.

In contrast to existing prediction models for growth (6–17), curve matching only requires HSDS data and can be used at any point in time during treatment. The accuracy of the prediction is presented by showing the variability of the growth curves of the matched patients, and the results are interpretable and explainable without adding significant workload to the clinical pathway. The matching technique enables HCPs to utilize their experience to implicitly match a new patient with similar patients they may have seen in the past. This can enhance, enrich, and assist the HCP’s memory of a comparable strategy, facilitating better-informed decisions based on more experience (21). It may also provide a source of reassurance for families on their child’s potential future growth, help manage expectations, and facilitate communication with the HCP during clinical visits.

Creating a visual representation of future growth can support an individualized approach to treatment to optimize growth outcomes. For example, in case of poor growth response due to sub-optimal adherence, the patient and HCP can visualize its impact on the patient’s HSDS or height later in treatment. This may help to better understand the consequences of missed r-hGH doses and may, as described above, facilitate the dialogue between patient and HCP to identify the potentially modifiable key factors associated with r-hGH sub-optimal adherence (26). It may also help pinpoint those patients and caregivers most likely to benefit from further education or participation in patient support programs (PSPs). Here too, visualized matching data could enhance the dialogue between patient/caregiver and PSP nurse.

### Findings

With curve matching and the availability of at least two measurements, the error SD for a one-year prediction was approximately 0.2, which corresponds to 1.1 cm, 1.3 cm, and 1.5 cm at 7 (before puberty), 11 (around puberty onset), and 15 years of age (during puberty), respectively. These values are comparable to the reported values obtained with previously developed, yet more complex, models. With respect to indication, our study found that matching with a larger sample of patients with almost similar growth responses (which is the case in GHD and SGA patients) improved the restriction of only matching with patients with a similar indication. Note, however, that when matching a new patient with both GHD and SGA patients, the dose of the matched patients does not provide information on dose management for the new patient. Moreover, one cannot match with patients with other indications such as Turner Syndrome, as we know that patients with Turner Syndrome have generally lower catch-up growth than patients with GHD or SGA.

### Digital solutions: current and future directions

The use of technology to support health and healthcare has grown rapidly in the last decade (27). For example, telemedicine platforms (28), clinical decision support within dashboard-based systems (29), as well as game-based interventions (30, 31) and augmented reality (32) have been integrated into education and assistive care in diabetes mellitus. Technology could also facilitate the earlier identification (33) and support of patients with growth disorders across their treatment journey. The easypod™ injection device, which wirelessly transmits recorded data from the device to the easypod™ connect system, provides an ‘internet of things’-based solution for the monitoring and understanding of adherence in patients with growth disorders (5). In principle, information on growth could also be added to this system. This provides the opportunity to use these real-world data for further analysis to gain insights and identify new opportunities to support HCPs and patients. Enhanced visualization or clinical decision support within a dashboard-based system providing timely knowledge and patient-specific information for HCPs, could play a considerable role in the management of growth disorders and treatment through additional and comprehensive support and monitoring. For example, a dashboard-based system with integrated prediction models for adherence (34, 35), persistence of use (36), and growth could identify patients with sub-optimal outcomes. Medical apps for mobile phones, such as growlink™ (part of the easypod™ connect ecosystem), can be used by patients and their families to monitor progress and provide educational information.

Curve matching, in combination with clustering techniques, could generate groups of patients with similar growth trajectories; for example poor, average, or good responders. These analyses offer an opportunity to generate digital personas (avatars) that a patient can identify with, as these avatars represent a similar growth response. These avatars could utilize behavioral change methodologies to motivate patients (they visualize that a low catch-up growth can lead to a significant reduced height later in treatment), improve adherence and ultimately optimize catch-up growth, as literature shows that greater identification translates into intrinsic motivated behavior (37). From a health economics perspective, there is also potential to compare predicted and actual outcomes at a broader population level to assess the medical value provided by an intervention. However, one needs to be aware that integration of curve matching within medical devices needs further investigation. The first step is to investigate the requirements for curve matching, such as: 1) are the data of the new patient accurately measured and correctly entered in the system, 2) are sufficiently strong matches available given the data of the patient, and 3) how can we support in interpreting the outcomes, especially if the matched growth curves are far apart or if a new measurement starts to deviate from the predicted growth curve. The second step is to capture end-users’ feedback. In recent years, much effort has been put into the application of new methodologies to capture end-users’ feedback when implementing digital innovations, including participatory research and design research. Such methodologies address the needs and perspectives of patients and HCPs for adjusting digital innovations to minimize adoption challenges. Important issues to take into account are that the advice should be interpretable and explainable, and that the outcomes should be clinically meaningful. Personalization is key, because the content should be relevant for each unique patient. The advice should also improve shared decision-making and, ultimately, have a positive impact on health outcomes. When the feedback is taken into account, the third step is to implement the digital innovations, by enabling HCPs to have access to all tools, information, and services that are needed. It is important that there is broad awareness and acceptance for new digital innovations among both patients and HCPs. An enhanced digital platform is then established as the basis for real-world evaluation of (determinants of) use and outcome, for example, in prospective clinical trials (fourth step). These steps have to be taken in order to use the curve matching technique in daily practice using an enhanced digital platform that can support patients in their treatment journey (38).

### Strengths and limitations

The key strengths of this study include the availability of large amounts of real-world growth data and the possibility to implement curve matching using these data within the easypod™ connect system or on a local system, if a software plug to communicate with a curve matching database is made available. Indeed, the availability of the easypod™ connect system offers an important prerequisite for translation into clinical practice.

Curve matching uses a simple algorithm that only compares the HSDS at certain time points with the HSDS of patients in the ‘Matching Database’ and selects those patients whose HSDS is closest to that of the new patient. This may simplify the technical integration of the matching algorithm. A limitation is that no other relevant parameters were taken into account for curve matching. The database should consist of a large amount of growth curves of patients in order to obtain good matches, especially if matched patients need to be found when the new patient is longer on treatment and has multiple HSDS to match with. However, since there is a strong cumulative increase in the number of patients using the easypod™ connect system, we expect the number of available growth curves to increase over time, which will enable stronger matches to be found, as well as matching with patients who have similar growth-related parameters as the new patient; for example, by selecting patients with similar age at start. Our previous research on the development of a growth prediction model with baseline parameters in a subset of our data has already shown that HSDS and age at start were highly statistically significant, and that sex, target height, r-hGH dose, puberty status (at baseline), birthweight SDS and region slightly improved the proportion of explained variance (from 71 to 72%) (39). Within the curve matching technique, there should be a balance between sufficient available patients for matching (more available patients imply better matches) and selecting a representative subgroup of patients (for example, with similar age at treatment start) for matching. Matching with a larger group outweighs matching with a smaller group based on factors that have only a small impact on the accuracy of the prediction. However, if some factors play a significant role, selecting subgroups or only providing the predictive value in patients who comply to selection criteria (for example, only prepubertal patients) is needed to apply this technique for individual patients in a clinical setting. Matching by puberty status during treatment may be of interest because our study shows that the predicted growth curve after 24 months of treatment was, on average, too low for patients aged ≥11 years at treatment start. Besides the aforementioned growth-related parameters, other important matching factors are the patients’ engagement to treatment expressed as adherence (mg injected/mg prescribed), motivation, activation, and satisfaction scores to GH treatment. Moreover, the underlying causes (from genetic to maternal) of intrauterine as well as permanent growth impairment in SGA may be of interest. Therefore, adding more parameters to facilitate matching may further improve the validity of curve matching, but only if a higher number of growth curves are available in order to find appropriate matches. Also, if parental height is available for all patients in the database, one can also match with HSDS-target height SDS to take the genetic effect into account.

If the number of available matches is low, privacy concerns, however, may pose a potential risk if a unique combination of personal data could lead to the matched personal data being identified, which is undesirable (21). The non-disclosure risk may limit the range of parameters that can be used to find matches (e.g. age at start of treatment truncated or in categories, region instead of country). Another possibility of curve matching is to also match on HSDS before treatment start to account for the growth velocity without receiving r-hGH. This may result in a better selection of patients with similar growth curves and may, therefore, improve the prediction of future growth. It may also present the opportunity to provide a good prediction at treatment start, rather than waiting up until the end of the first three months of treatment. However, such data are currently not available. Another limitation is the fact that, while the vast majority of patients were treatment-naive, for some this may not have been the case. It is known that catch-up growth is generally higher in the first year of treatment compared with later time points. If such information can be added, then a more homogeneous set of patients could be selected, which may also improve the validity of curve matching. Another limitation is that the WHO growth references (22, 23) were used for all patients to calculate HSDS. An improvement may be to possibly use country-specific growth references. Lastly, another limitation, which also applies to general prediction models, is that height should be accurately measured and entered into the system. For example, it is unknown if the second measurement in **Figure 3** is accurate, but it is clearly visible that this measurement has a major impact on the prediction.

## Conclusions

Curve matching is a valid technique that provides an interpretable and explainable visualization and prediction of future growth in patients with GHD and those born SGA. Future analysis utilizing more data has the potential to enhance this technique to find even stronger matches. Further external validation of this technique in predicting growth is recommended. Curve matching has the potential to integrate within a digital ecosystem to enhance and improve the care of children with growth disorders. We expect that curve matching could also be relevant for predicting outcomes in children with other growth disorders (for example Turner Syndrome) as well as in other areas of healthcare, such as matching patients with diabetes mellitus with similar blood glucose levels.

## Data availability statement

Any requests for data by qualified scientific and medical researchers for legitimate research purposes will be subject to Merck’s Data Sharing Policy. All requests should be submitted in writing to Merck’s data sharing portal https://www.merckgroup.com/en/research/our-approach-to-research-and-development/healthcare/clinical-trials/commitment-responsible-data-sharing.html. When Merck has a co-research, co-development, or co-marketing or co-promotion agreement, or when the product has been out-licensed, the responsibility for disclosure might be dependent on the agreement between parties. Under these circumstances, Merck will endeavour to gain agreement to share data in response to requests.

## Ethics statement

Treatment with easypod™ was conducted according to local practice. This real-world, observational, retrospective analysis of easypod™ data was performed in accordance with the informed consent form, signed by caregivers of children and adult patients materializing their agreement for data collection, storage, and use of their pseudonymized data to create aggregated statistical and general adherence reports. The research protocol (registration no. 2021-115) was submitted to The Netherlands Organization for Applied Scientific Research (TNO) Institutional Review Board. The board approved the research proposal. In its deliberations, the board considered the research design and privacy aspects, as well as the ethical aspects and the burden and risks to the research participants. The ECOS was conducted in accordance with the principles of the Declaration of Helsinki, Good Clinical Practice (ICH-GCP E6) guidelines and applicable national legal and regulatory requirements. Written informed consent was obtained from patients (or their parent/guardian) prior to study enrollment. The study was approved by local ethical committees for the centers in each country.

## Author contributions

PD provided substantial contributions to the research design, analysis and interpretation of data, drafting the paper, and approval of the submitted and final versions. EK provided substantial contributions to the interpretation of data, revising the paper critically, and approval of the submitted and final versions. LA provided substantial contributions to preparing the data, interpretation of data, revising the paper critically, and approval of the submitted and final versions. All authors contributed to the article and approved the submitted version.

## Funding

Merck (CrossRef Funder ID: 10.13039/100009945).

## Acknowledgments

Medical writing assistance was provided by Amy Evans of inScience Communications, Springer Healthcare Ltd, UK, and was funded by Merck Healthcare KGaA, Darmstadt, Germany, in accordance with Good Publication Practice (GPP3) guidelines (http://www.ismpp.org/gpp3). We thank Allan Jones of Merck Healthcare KGaA, Darmstadt, Germany for providing important intellectual content and creating **Figure 1**. We thank Professor Paul Dimitri, of The Academic Unit of Child Health, Sheffield Children’s NHS Foundation Trust, Sheffield, UK for reviewing this manuscript.

## Conflict of interest

PD has a consultancy agreement with Merck Healthcare KGaA, Darmstadt, Germany. LA is an employee of Ares Trading S.A., Eysins, Switzerland (an affiliate of Merck KGaA). EK is an employee of Merck Healthcare KGaA, Darmstadt, Germany and holds shares in the company.

The analysis was performed by PD from TNO. TNO is an independent RTO (*Regeling Tewerkstelling Officieren*) from The Netherlands founded by law. TNO’s professionals put their knowledge and experience to work in creating smart solutions to complex issues. These innovations help to sustainably strengthen social wellbeing and industrial competitiveness. The core values of TNO are integrity, independence, professionalism, and engagement with society. TNO’s Code of Conduct, published on the website of TNO (https://www.tno.nl/en/about-tno/missionand-strategy/tno-code), contains a chapter on scientific integrity. Research is conducted without any undue influence from commercial or other interests. Merck provided access to the data. The manuscript was reviewed according to Merck’s publication procedures.

## Publisher’s note

All claims expressed in this article are solely those of the authors and do not necessarily represent those of their affiliated organizations, or those of the publisher, the editors and the reviewers. Any product that may be evaluated in this article, or claim that may be made by its manufacturer, is not guaranteed or endorsed by the publisher.
